# Extraperitoneal urine leak after renal transplantation: the role of radionuclide imaging and the value of accompanying SPECT/CT - a case report

**DOI:** 10.1186/1471-2342-10-23

**Published:** 2010-10-20

**Authors:** Hongju Son, Sherif Heiba, Lale Kostakoglu, Josef Machac

**Affiliations:** 1 The Division of Nuclear Medicine, Department of Radiology, The Mount Sinai School of Medicine, New York, NY, USA

## Abstract

**Background:**

The differentiation of the nature of a fluid collection as a complication of kidney transplantation is important for management and treatment planning. Early and delayed radionuclide renography can play an important role in the evaluation of a urine leak. However, it is sometimes limited in the evaluation of the exact location and extent of a urine leak.

**Case Presentation:**

A 71-year-old male who had sudden anuria, scrotal swelling and elevated creatinine level after cadaveric renal transplantation performed Tc-99 m MAG3 renography to evaluate the renal function, followed by an ultrasound which was unremarkable. An extensive urine leak was evident on the planar images. However, an exact location of the urine leak was unknown. Accompanying SPECT/CT images confirmed a urine leak extending from the lower aspect of the transplant kidney to the floor of the pelvic cavity, presacral region and the scrotum via right inguinal canal as well as to the right abdominal wall.

**Conclusions:**

Renal scintigraphy is very useful to detect a urine leak after renal transplantation. However, planar imaging is sometimes limited in evaluating the anatomical location and extent of a urine leak accurately. In that case accompanying SPECT/CT images are very helpful and valuable to evaluate the anatomical relationships exactly.

## Background

The most effective primary treatment of chronic renal failure is renal transplantation [1-3]. Most surgical complications involve either the wound or one of the three anastomoses (renal artery, renal vein, or ureter). A fluid collection is a common complication after kidney transplantation [1,4-6]. Causes of fluid collection include: lymphocele, urine leak, hematoma and seroma. Fluid collections can be asymptomatic, or may be associated with swelling and pain at the site of the allograft, wound drainage, swelling of the ipsilateral lower extremity, and occult blood loss [4,5]. Approximately two thirds of early urologic complications (urine leaks and obstruction) are apparent in the first month after transplantation [6]. It is often difficult to distinguish the signs and symptoms of urinary extravasation from those of rejection or obstructive uropathy. To aid in the early and definitive diagnosis ultrasound scanning, isotope renal scanning, magnetic resonance urography, antegrade or retrograde urography, and/or cystography are performed [7]. An ultrasound plays a crucial role in diagnosis of postoperative complications, often directly revealing fluid collections, dilated collecting systems, and vascular stenosis [1]. However, an ultrasound cannot differentiate the nature of the collection easily, and such differentiation is important for management and treatment planning. Early and delayed radionuclide renography can play an important role in the evaluation of a urine leak [8]. However, this is sometimes limited in the evaluation of the exact location of a urine leak. Our case illustrates the importance of SPECT/CT imaging in the evaluation of the location and extent of a urine leak which are not sufficiently revealed on planar renal scintigraphy.

## Case Presentation

**Figure 1:**
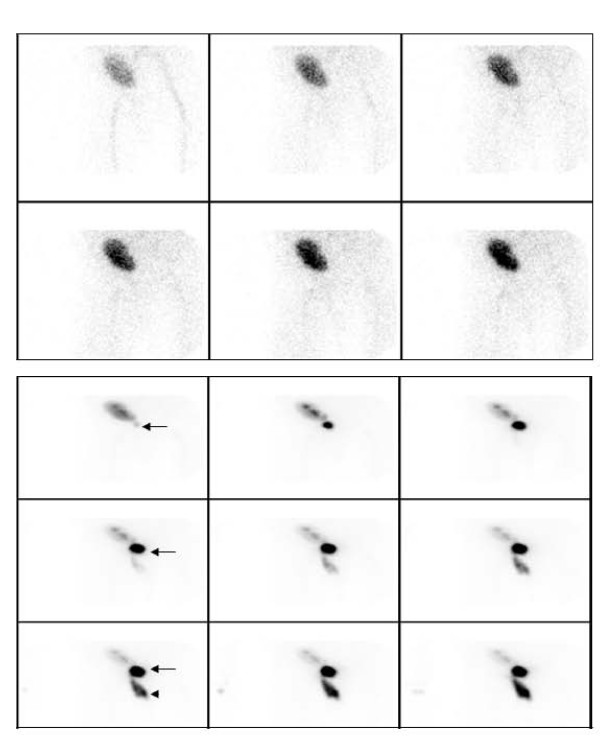
**Renal scintigraphy performed immediately after the patient had sudden onset of scrotal swelling and anuria**. (A) Initial blood flow images (2 sec/frame) show normal renal perfusion. Here the summed flow images are shown. (B) On subsequent 30-min functional images were obtained. Here the summed functional images demonstrate the transplant kidney showing normal perfusion and function with excretion of radiotracer into the renal collecting system and a focal collection mimicking the urinary bladder (arrows). There is a vertically oriented accumulation of radioactivity inferior to the pelvis (arrow head).

**Figure 2:**
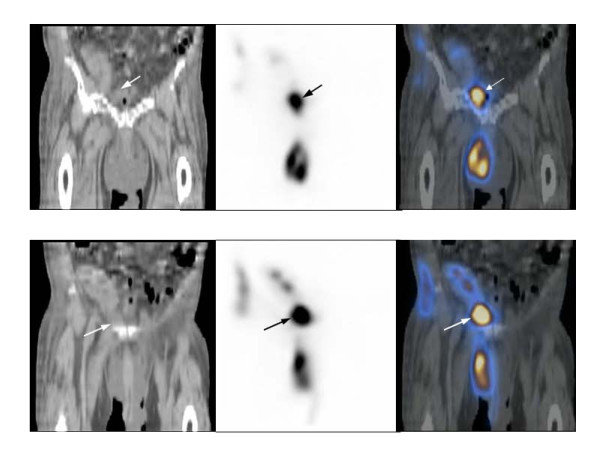
**Coronal images of SPECT/CT of pelvis**. (A) The images show a radiotracer accumulation demonstrating a urine leak (arrows) in the right pelvic cavity just next to the urinary bladder and in the scrotum. A Foley catheter balloon is located in the very contracted urinary bladder. (B) More anterior coronal slice shows a urine leak (arrows) in the right pelvic cavity just below the transplanted kidney extending to the scrotum and the right abdominal wall.

**Figure 3:**
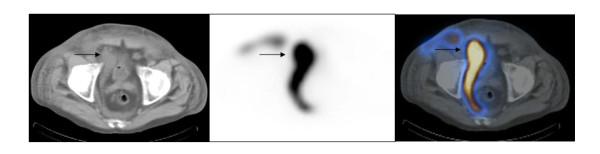
**Axial images of SPECT/CT of pelvis**. The images show a urine leak (arrows) in the right pelvic cavity next to the urinary bladder extending to the right inguinal canal and abdominal wall anteriorly and the presacral region posteriorly.

**Figure 4:**
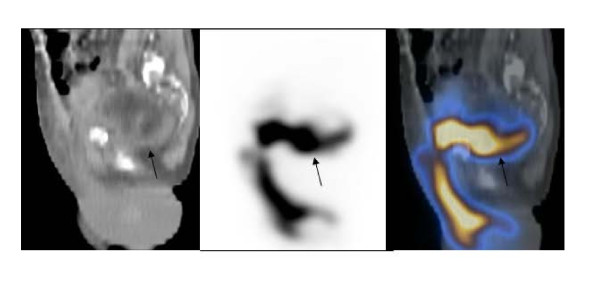
**Saggital images of SPECT/CT of pelvis**. The images show an accumulation of radiotracer (arrows) in the pelvic floor extending to the presacral region posteriorly, and subcutaneous prepubic region extending to the sacrum inferiorly, and the anterior abdominal wall upwardly.

The patient was a 71-year old male with history of diabetes mellitus, hypertension, end- stage of renal disease on hemodialysis. He underwent cadaveric renal transplantation to the right lower abdomen without complications. The early postoperative course was uneventful. The patient maintained the adequate urine output. The creatinine level decreased to 1.0. However, on the ninth postoperative day, he experienced sudden pain and swelling in his scrotum, with a drop in a urine output to nearly zero. His creatinine levels abruptly climbed from a nadir of 0.9 to 3.4 over 4 days. The patient's scrotum was markedly dilated with scrotal cutaneous urine leaks. The patient underwent an ultrasound examination, showing the normal looking kidney in the right lower quadrant of the abdomen with normal perfusion and no evidence of hydronephrosis or a perinephric fluid collection. On the next day, the patient underwent Tc-99 m MAG3 radionuclide renography which showed normal renal flow and function with a complete drainage of activity from the renal collecting system to what looked like the urinary bladder. In addition, there was an evident urine leak and an accumulation of radiotracer activity in the pelvic floor extending below the pelvic floor. However, the exact location of the urine leak was unknown (Figure 1A and 1B). SPECT/CT imaging of the pelvis was obtained to evaluate the exact location and extent of the urine leak. The low dose noncontrast CT was obtained for the purpose of anatomic co-registration with the SPECT images. The obtained SPECT/CT images showed diffuse extensive radiotracer activity extending from just below the transplant kidney to the right pelvic cavity next to the urinary bladder, presacral region, right prepubic region and the scrotum and penis which were markedly dilated as well as into the right abdominal wall (Figure 2A and 2B, Figure 3, Figure 4). The urinary bladder was totally contracted with a Foley catheter, from which no radioactivity drained. The structure looked like the urinary bladder on planar renography was not the real urinary bladder after all, but an accumulation of urine next to the urinary bladder (Fig. 2A). An emergency operation was undergone. It revealed a ureteral leak secondary to ischemic necrosis of the distal transplanted ureter. A take-down ureteroneocystostomy and cystorrhaphy, right native ureteroureterostomy with insertion of a stent in the transplant ureter, and right native ureteroureterostomy to the transplant UVJ were performed. The postoperative course was uneventful.

Ureteral obstruction and extravasation of urine occur in less than 5% of renal transplants and are usually manifest more than one week after surgery [4,6]. A posttransplant urine leakage usually requires invasive treatment by either interventional radiology or early surgery [1]. Early surgical exploration with ureteral reimplantation is indicated for very early leaks, large leaks, or leaks that do not respond to conservative measures.

The most common cause of ureteral obstruction or a urinary leak is ischemia of the ureter or renal pelvis. Native ureters have a triple blood supply from blood vessels from the renal pedicle, the adjacent lumbar arteries and the urinary bladder. The last two vascular supplies are lost when the kidney is removed from the donor. The vascular supply to the ureter from the renal pedicle is tenuous, at best, and easily damaged. With ureteral ischemia the ureter becomes fibrotic and obstructed or breaks down and leaks.

It usually arises at the anastomotic site [4]. Causes other than ischemia include undue tension created by the short ureter and direct surgical trauma to the ureter (usually at the time of procurement). Symptoms include fever, pain, swelling at the graft site, increased creatinine level, decreased urine output, and a cutaneous urinary drainage.

The diagnosis of a urine leak after renal transplantation is often made by a combination of clinical findings and imaging studies [8]. The laboratory findings may not be specific because serum creatinine values do not provide a consistent indication of a leak, as they do in cases of obstruction. A leak may result in some systemic reabsorption of urine and hence elevated serum creatinine often mimicking obstruction. Given the difficulty of a clinical diagnosis, imaging studies are therefore necessary to substantiate the presence of urine leakage [4,9]. Sonography and nuclear renography are the most commonly used imaging studies to diagnose urine leaks [10-12]. Although sonography is excellent at suggesting the possibility of leakage, the detection of peritransplant fluid by sonography is not specific for a leakage. Although scintigraphic detection of urine leaks has been well documented and nuclear renography is more helpful in the diagnosis of larger leaks, it is dependent on good renal function and limited by poor excretion of the radionuclide in the cases of poor renal function [10-12].

Our case showed normal renal flow and function of transplanted kidney with a definite urine leak just below the transplant kidney into the pelvic cavity on the renal scintigraphy. We interpreted a round lesion in the midline of pelvic cavity just below the transplanted kidney as a partial filling of the urinary bladder. The exact evaluation of the location of a urine leak was not possible. The obtained SPECT/CT images showed diffuse extensive radiotracer activity to suggest a urine leak just below the transplant kidney extending to the right pelvic cavity next to the urinary bladder, presacral region, right prepubic region, and the scrotum and penis which were markedly dilated as well as into the right abdominal wall. In other words, SPECT/CT images localized the anatomical location and extent of leaked urine accurately as well as its anatomical relationship with the transplant kidney. The extent of a urine leak was much greater on SPECT/CT images than that was considered on planar imaging. In addition, the structure mimicking the urinary bladder was not the urinary bladder, but an accumulation of urine located in the right pelvic cavity next to the urinary bladder and right inguinal region. We found that SPECT/CT imaging is very useful to localize the urine leak and its extent accurately in the case where it is not possible to see its exact anatomical relationship with the adjacent structures and location on planar renal scintigraphy.

## Conclusions

Renal scintigraphy is very useful to detect a urine leak after renal transplantation. However, planar imaging is sometimes limited in evaluating the anatomical location and extent of a urine leak accurately. In that case accompanying SPECT/CT images are very helpful and valuable to evaluate the anatomical relationships exactly.

## Competing interests

The authors declare that they have no competing interests.

## Authors' contributions

HS participated in the design of the case report and wrote the manuscript and designed the format of figures. SH participated in the sequence alignment. LK participated in the sequence alignment and collected and provided references. JM conceived of the study and participated in its design and coordination. All authors read and approved the final manuscript.

## Pre-publication history

The pre-publication history for this paper can be accessed here:

http://www.biomedcentral.com/1471-2342/10/23/prepub
